# Factors associated with microbiological non-compliance in ready-to-eat foods: A seven-year analysis of official food safety surveillance data in a tropical low- and middle-income country

**DOI:** 10.1371/journal.pgph.0006492

**Published:** 2026-10-05

**Authors:** L. Marcela Montilla Rodríguez, Jorge Alejandro Obando Bastidas, Hugo Andrés Rodríguez Fajardo, Astrid Magaly Mora López, Shirley Viviana Tique Aragón, Dora Patricia Rozo Cruz, Edgar Calderón Vega, Norma Cristina Pavas Escobar, Laura Nathalia Obando Vargas, Jean Paul Farfán, Marina Sthella Gonzalez Robayo

**Affiliations:** 1 GRIVI Research Group, Faculty of Medicine, Universidad Cooperativa de Colombia, Villavicencio, Colombia; 2 GISSME Research Group, Secretaría de Salud del Meta, Villavicencio, Colombia; 3 GEDOVAZ Research Group, Faculty of Business Administration, Universidad Cooperativa de Colombia, Villavicencio, Colombia; PLOS: Public Library of Science, UNITED STATES OF AMERICA

## Abstract

Food safety is an important component of global public health, particularly in low- and middle-income countries (LMICs). This study aimed to identify factors associated with microbiological non-compliance in ready-to-eat (RTE) foods using seven years of official surveillance data from the Colombian Orinoquía region. RTE foods were defined as foods intended for immediate consumption without additional cooking or thermal processing. A retrospective analysis was conducted on 1,868 samples collected between 2017 and 2023, of which 1,846 were included in inferential analyses. Microbiological non-compliance, defined as failure to meet at least one regulatory microbiological criterion, was the primary outcome. Associations were assessed using multivariable logistic regression, complemented by descriptive and exploratory analyses, including heatmaps and principal component analysis. Microbiological non-compliance was identified in 39.5% of the evaluated samples. Hygiene indicators predominated, particularly total coliforms (29.0%), aerobic mesophilic bacteria (12.7%), and *Escherichia coli* (12.3%), whereas foodborne pathogens were infrequently detected. Raw or highly handled foods had higher odds of microbiological non-compliance than thermally processed foods (OR = 8.40; 95% CI: 6.59–10.72). Higher odds were also observed for samples from rural or dispersed municipalities (OR = 1.31; 95% CI: 1.01–1.70) and samples collected in 2020 (OR = 1.78; 95% CI: 1.16–2.74). Exploratory analyses revealed heterogeneous microbiological profiles across temporal, territorial, operational, and food-related dimensions, with a consistent predominance of hygiene indicators. Overall, microbiological non-compliance was mainly associated with food matrix type, territorial characteristics, and sampling year. The predominance of hygiene indicators supports incorporating hygienic quality and process control alongside pathogen detection into microbiological surveillance. These findings provide evidence to support risk-based food safety surveillance and targeted prioritization strategies in tropical low- and middle-income settings.

## 1. Introducción

Food safety is a fundamental pillar of global public health due to its role in preventing foodborne diseases (FBD) and protecting population health. Globally, FBD affect more than 600 million people each year and cause approximately 420,000 deaths, with a higher burden in low- and middle-income countries where structural limitations persist in sanitation infrastructure, food safety regulation, and surveillance systems [1]. In these settings, environmental conditions, food handling practices, and limitations in hygienic control contribute to microbiological contamination and non-compliance with microbiological standards established for food safety surveillance.

In this study, ready-to-eat (RTE) foods were defined as foods prepared and intended for immediate consumption without additional cooking or thermal processing. The study focused on foods prepared or handled in establishments included in the official food safety surveillance program, excluding commercially packaged industrialized foods. These foods represent an important public health concern because microorganisms introduced during preparation, handling, or distribution may reach consumers directly. According to the World Health Organization (WHO), the burden of foodborne diseases is greatest in low- and middle-income countries (LMICs), where tropical climates, rapid urbanization, and operational challenges may contribute to microbiological contamination of foods. In this context, official food safety surveillance plays a critical role in monitoring microbiological compliance, identifying food categories and contextual factors associated with non-compliance, and supporting evidence-based risk management strategies. Generating this type of evidence in tropical regions such as Meta, Colombia, may provide valuable information for strengthening surveillance programs in other LMICs facing similar environmental and operational conditions.

From a global health perspective, food safety in low- and middle-income countries (LMICs) is influenced by multiple contextual factors, including access to safe water, sanitation infrastructure, hygienic practices, and the capacity of food safety surveillance systems. These conditions may affect the microbiological quality of ready-to-eat foods, particularly in informal food environments such as street vendors and small-scale food establishments, where operational challenges are more common. Consequently, official microbiological surveillance plays a fundamental role in monitoring compliance with microbiological standards, identifying priority food categories, and generating evidence to support risk-based food safety interventions. Such efforts contribute to strengthening food safety systems and support progress toward the Sustainable Development Goals (SDGs), particularly SDG 2 (Zero Hunger), SDG 3 (Good Health and Well-being), and SDG 6 (Clean Water and Sanitation) [1,2>].

Within this context, the microbiological quality of ready-to-eat foods is commonly assessed through hygienic indicators that reflect handling, processing, and storage conditions. Consequently, contamination introduced during preparation, processing, or distribution may affect the microbiological quality of these foods. Indicators such as total coliforms, E*scherichia coli*, aerobic mesophilic bacteria, molds, and yeasts provide information on hygienic failures, inadequate water quality, cross-contamination, and insufficient temperature control [3–6]. Their presence is considered a marker of poor hygienic practices and an indirect indicator of microbiological quality and process control.

The official food safety surveillance system routinely evaluated hygienic indicators, including *Escherichia coli*, total coliforms, aerobic mesophilic bacteria, molds, and yeasts, as well as selected foodborne pathogens, including *Salmonella* spp., *Listeria monocytogenes, Bacillus cereus*, and coagulase-positive *Staphylococcus*, according to the microbiological criteria established by the national surveillance program [7–10]. These microbiological parameters were used to assess compliance with the regulatory microbiological criteria established for food safety surveillance.

Environmental conditions play a significant role in shaping patterns of microbiological contamination. In tropical climates, characterized by high temperatures and humidity, microbial growth is favored. Additionally, heterogeneity in food handling practices, variability in water quality, and limitations in the cold chain may contribute to microbiological contamination and non-compliance with microbiological standards, particularly in particularly in raw or highly handled foods such as juices and salads [6,11,12].

In Colombia, the Orinoquía region—particularly the Meta department—presents environmental, productive, and operational conditions that may influence microbiological contamination in foods. This region is characterized by marked urban–rural heterogeneity and rapid agro-industrial growth. However, available scientific evidence remains limited and is largely restricted to localized descriptive studies, with few analytical studies using official surveillance data to identify factors associated with microbiological non-compliance in ready-to-eat foods. This knowledge gap highlights the need for studies capable of supporting evidence-based food safety surveillance in tropical settings of low- and middle-income countries [1,2].

In this context, the aim of this study was to identify factors associated with microbiological non-compliance in ready-to-eat foods in the Meta department (Colombia) using official food safety surveillance data collected between 2017 and 2023. Microbiological non-compliance, defined as failure to meet at least one regulatory microbiological criterion, was used as the primary outcome. To address this objective, microbiological indicators were analyzed together with temporal, territorial, and food-related variables using a sequential analytical approach in which multivariable logistic regression served as the primary inferential analysis to identify factors independently associated with microbiological non-compliance. The findings provide evidence to support risk-based food safety surveillance and prioritization strategies in tropical settings.

## 2. Materials and methods

### 2.1 Ethics statement

This study was based exclusively on the secondary analysis of anonymized data derived from routine public health surveillance in the Meta department. Samples were obtained during mandatory sanitary inspections, without involving direct intervention on individuals or establishments, nor procedures requiring informed consent. The data were accessed for research purposes in August 2024. All records used had been previously de-identified by the Public Health Laboratory and contained no personal or commercial information, nor direct or indirect identifiers. The authors did not have access to any information that could identify individual participants or establishments at any stage of the study, either during or after data collection. According to national regulations and international standards, including those established by the World Health Organization (WHO) and the Declaration of Helsinki (2013), analyses of de-identified data from official surveillance systems were classified as exempt from formal ethical review

### 2.2 Study design

A retrospective observational study was conducted based on the analysis of official results from the Public Health Laboratory (PHL) of the Meta department, corresponding to microbiological surveillance of ready-to-eat (RTE) foods carried out between 2017 and 2023. These datasets were generated within the framework of routine inspection, surveillance, and control activities.

### 2.3 Study area and target population

The study was conducted in the Meta department, Colombia, a tropical region belonging to the Colombian Orinoquía. It is characterized by marked productive and commercial heterogeneity, ranging from extensive livestock systems to urban areas with establishments dedicated to the preparation, handling, and commercialization of RTE foods.

Samples were collected from 32 municipalities (29 within the Meta department and 3 from neighboring departments), included due to their geographical proximity and operational integration with the Meta Public Health Laboratory. The theoretical minimum sample size was estimated at 384 samples using Cochran’s formula for estimating a proportion in a large population, assuming a 95% confidence level, an expected prevalence of 50%, and a 5% margin of error. However, the official surveillance system included a total of 1,868 samples, substantially exceeding the minimum required sample size and increasing the precision of the estimates and the robustness of the analyses.

### 2.4 Inclusion and exclusion criteria

Samples of foods prepared and intended for immediate consumption without additional cooking or thermal processing before consumption were included. Eligible samples had complete traceability and were collected under aseptic conditions as part of the official food safety surveillance program. Exclusion criteria comprised packaged industrialized foods, raw materials, samples without adequate cold chain maintenance, and samples with insufficient volume.

### 2.5 Data sources and data management

Samples were collected as part of the routine official food safety surveillance program by trained environmental health technicians following national inspection, surveillance, and control procedures. Food establishments were selected according to the monthly surveillance plan established by the departmental health authority. Samples were transported to the Public Health Laboratory (PHL) under cold-chain conditions following the laboratory chain-of-custody procedures. Upon receipt, samples were evaluated for integrity, labeling, temperature, and compliance with the laboratory acceptance criteria. Samples were maintained under the storage conditions established for each food matrix, as defined in the official laboratory procedures, and processed within 24 h of collection according to standardized procedures and official methodologies for the microbiological analysis of foods. Analytical results were extracted from the consolidated PHL databases together with operational information recorded in the sampling forms, including year of collection, sample temperature, municipality of origin, type of establishment, food matrix, and microbiological results. Prior to analysis, all records were anonymized by removing any information that could allow the direct or indirect identification of establishments or individuals, thereby ensuring data confidentiality.

### 2.6 Study variables

#### 2.6.1 Microbiological indicators.

Microbiological criteria were defined based on national guidelines for food safety surveillance in Colombia, as applied in routine laboratory practice, in accordance with standards used by the National Institute for Food and Drug Surveillance (INVIMA). Microbiological analyses were performed using standardized laboratory procedures based on internationally recognized reference methods, including ISO standards, AOAC Official Methods, FDA methodologies, and validated automated TEMPO methods, as appropriate for each microbiological parameter. Detailed information on the analytical methods, culture media, incubation conditions, detection procedures, reporting units, and reference standards is provided in S1 Table. The evaluation included selected foodborne pathogens—*Salmonella* spp., *Listeria monocytogenes*, coagulase-positive *Staphylococcus*, and *Bacillus cereus*—as well as hygienic indicators—*Escherichia coli*, total coliforms, aerobic mesophilic microorganisms, molds, and yeasts. Microbiological acceptability criteria were established on the basis of regulatory limits for 10 g or 10 mL of sample: mesophilic microorganisms (<10,000 CFU/g or mL), total coliforms (<10 MPN/g or mL), *E. coli* (<10 MPN/g or mL), coagulase-positive *Staphylococcus* (<100 CFU/g), *Bacillus cereus* (<100 CFU/g), and molds and yeasts (<10 MPN/g or mL). Additionally, detection of *Salmonella* spp. and *Listeria monocytogenes* in 25 g of sample was considered a rejection criterion. Because quantitative microbial concentrations were not consistently available in a standardized and directly comparable format across all microbiological parameters and surveillance years, the analyses were based on compliance with the applicable regulatory microbiological criteria and, for descriptive purposes, on the observed proportion of positive samples for each microbiological parameter. Based on these thresholds, samples were dichotomously classified as microbiologically compliant or non-compliant. Microbiological non-compliance was defined as failure to meet at least one microbiological criterion, either by exceeding the applicable regulatory limit or by detection of a pathogen.

#### 2.6.2 Contextual variables.

Contextual variables included temporal patterns (2017–2023), municipal distribution, food matrix type, and type of establishment.

### 2.7 Statistical analysis

During the study period, 1,868 RTE food samples were received. Of these, 1,846 had a valid final microbiological assessment and were included in the analyses, whereas 22 samples without a valid final microbiological assessment were considered non-evaluable for the primary outcome. These samples were excluded from the calculation of microbiological compliance and non-compliance proportions and from inferential analyses aimed at identifying factors associated with microbiological non-compliance. The primary outcome was coded as a dichotomous variable (0 = microbiologically compliant; 1 = microbiologically non-compliant).

Microbiological non-compliance, defined as failure to meet at least one regulatory microbiological criterion, was established as the primary outcome of the study. This binary outcome (compliant vs. non-compliant) was used in the bivariate and multivariable inferential analyses. In parallel, the proportion of samples testing positive for each microbiological parameter was examined exclusively for descriptive and exploratory purposes, including heatmaps and principal component analysis (PCA). These exploratory analyses were used to characterize patterns in the microbiological results and were not interpreted as measures of microbiological risk or as microorganism-specific determinants of non-compliance.

Microbiological non-compliance, defined as failure to meet at least one regulatory microbiological criterion, was established as the primary outcome of the study. This binary outcome was used in the bivariate and multivariable inferential analyses. In parallel, the proportion of samples testing positive for each microbiological parameter was examined exclusively for descriptive and exploratory purposes, including heatmaps and principal component analysis (PCA). These exploratory analyses were used to characterize patterns in the microbiological results and were not interpreted as measures of microbiological risk or as microorganism-specific determinants of non-compliance.

The analysis was structured at three levels: descriptive, bivariate, and multivariable. Descriptive analyses were used to characterize the distribution of microbiological results and study variables, whereas inferential analyses were used to identify factors associated with microbiological non-compliance. Categorical variables were summarized using absolute (n) and relative (%) frequencies.

Categorical variables were recoded and grouped according to operational and epidemiological criteria to improve model stability and epidemiological interpretability. The sampling year variable was grouped into three periods (2017–2019, 2020, and 2021–2023). The municipality variable was classified into three categories: Villavicencio (departmental capital and largest urban center), intermediate municipalities (Acacías, Granada, Cumaral, Restrepo, Puerto López, San Martín, and Puerto Gaitán), and rural/dispersed municipalities (Mesetas, San Carlos de Guaroa, Cabuyaro, El Castillo, Fuentedeoro, Mapiripán, Puerto Lleras, Barranca de Upía, Cubarral, Lejanías, El Calvario, El Dorado, Puerto Rico, San Juanito, Vistahermosa, Puerto Concordia, San Juan de Arama, Medina, Guayabetal, Paratebueno, Uribe, and La Macarena).

The type of establishment was grouped into five categories: food service (restaurants, cafeterias, street vendors, and public markets), food operators (food production and handling establishments), institutional (educational institutions, childcare centers, penitentiary centers, nursing homes, and hospitals), distribution/logistics (supermarkets, transport, and distributors), and others (military and household settings). The food matrix type was classified into five groups according to processing and handling characteristics: thermally processed foods (fish, chicken, meat, eggs, hamburgers, arepas, rice, empanadas, sausage, beans, stew, potato, and pasta); raw or highly handled foods (juices, salads, and salpicon); liquid preparations (broths/soups, coffee/hot chocolate, oatmeal, and masato); dairy and sweet products (milk, desserts, custard, cheese, and panela); and other less frequently sampled foods (water, salt, and ham).

Subsequently, a bivariate analysis was performed using Pearson’s chi-square test to assess the association between independent variables and microbiological non-compliance. Odds ratios (OR) and their corresponding 95% confidence intervals (95% CI) were calculated. Statistical significance was defined as p < 0.05.

For the multivariable analysis, a binary logistic regression model was fitted using maximum likelihood estimation and a purposeful variable selection approach. Variables with p-values < 0.20 in the bivariate analysis were considered for inclusion, together with those of epidemiological relevance, to avoid excluding potential confounders. Severe multicollinearity was identified in the initial model (condition index > 30), particularly among variables related to food matrix type. To address this, the variable was reparametrized by using thermally processed foods as the reference category and including the corresponding dummy variables for the remaining food matrix categories. This adjustment improved model stability, reducing variance inflation factors (VIF) to < 2.0 and ensuring parsimonious and interpretable estimates. Model fit was assessed using the Hosmer–Lemeshow test. Explanatory capacity was evaluated using Cox and Snell R^2^ and Nagelkerke R^2^, while discriminatory performance was assessed based on the proportion of correctly classified cases. The final model was evaluated for goodness-of-fit, multicollinearity, and epidemiological plausibility.

In addition, exploratory analyses were conducted using heatmaps to describe patterns in microbiological results according to temporal, geographic, and operational dimensions, as well as principal component analysis (PCA) to explore patterns of association among microorganisms, municipalities, and food matrices. For the temporal heatmap (Fig 1), the annual proportion of samples testing positive for each microbiological parameter was standardized separately across years using z-scores calculated from the corresponding mean and standard deviation. The observed proportions (%) were retained as cell labels, whereas the color scale represented the standardized values. Years were displayed chronologically and were not subjected to hierarchical clustering. In contrast, the operational heatmap by establishment type (S1 Fig) and the geographic heatmap by municipality (S2 Fig) displayed the observed proportions of positive samples without standardization and were used descriptively to illustrate variation across establishment types and municipalities, respectively. These analyses were considered descriptive and exploratory and were not used to infer microorganism-specific determinants of microbiological non-compliance.

**Fig 1:**
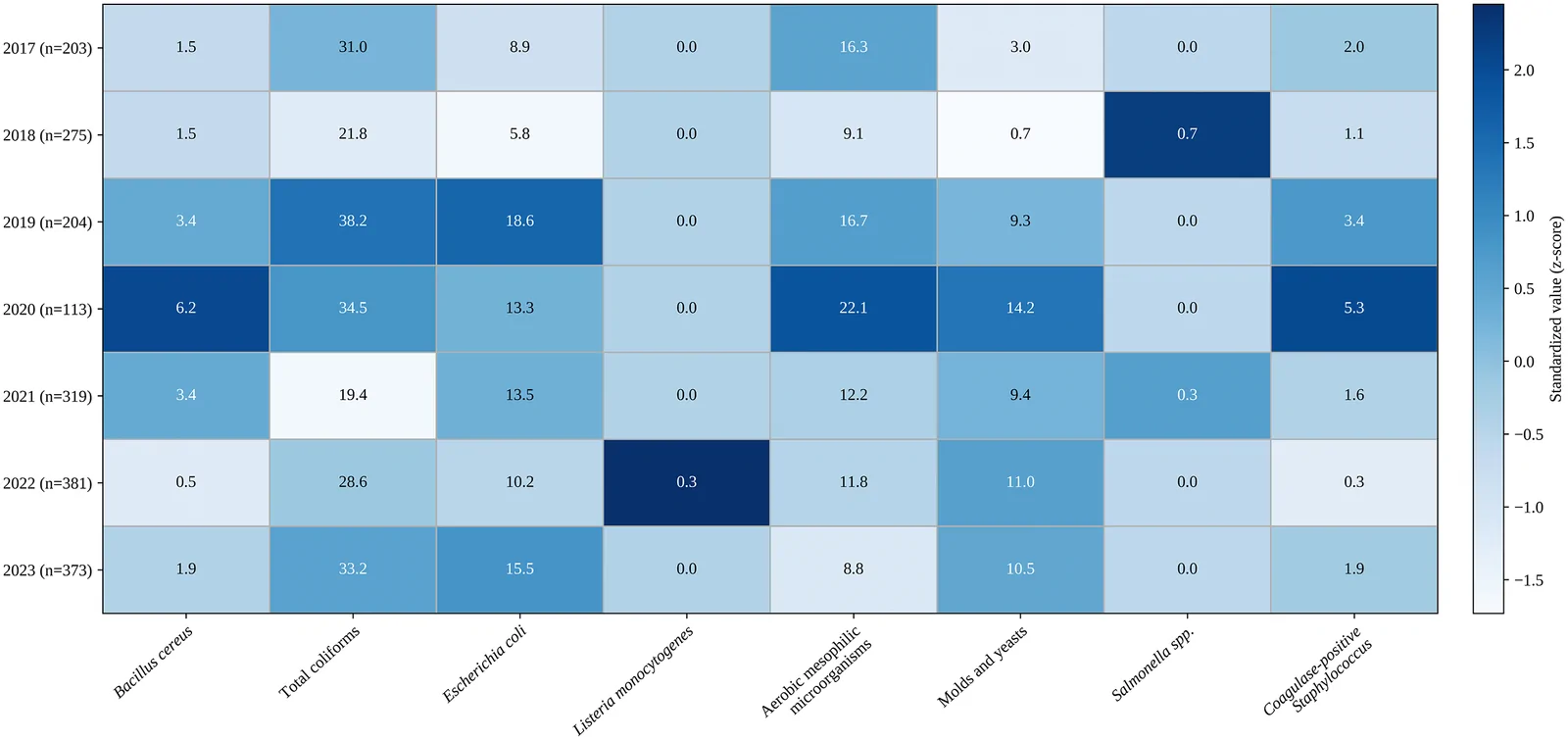
Temporal patterns in the proportion of samples positive for microbiological parameters in ready-to-eat foods, 2017–2023. Cell values represent the observed annual proportion of positive samples (%). Color intensity represents standardized values (z-scores) calculated separately for each microbiological parameter across years using the corresponding mean and standard deviation; darker shades indicate values relatively higher than the parameter-specific mean, whereas lighter shades indicate values relatively lower than the mean. Years are displayed chronologically and were not subjected to hierarchical clustering.

Statistical analyses were performed using R version 4.5.0 (R Core Team, 2023) and IBM SPSS Statistics version 27. The packages dplyr, tidyr, ggplot2, car, FactoMineR, factoextra, and pheatmap were used.

### 2.8 Use of generative artificial intelligence

ChatGPT (OpenAI) was used during manuscript revision as a language-editing and writing-support tool to improve English grammar, clarity, readability, and consistency of terminology. The tool was not used to generate study data, conduct microbiological or statistical analyses, determine study outcomes, or independently formulate scientific conclusions. All AI-assisted content was critically reviewed and verified by the authors against the original surveillance data, statistical results, and study methodology. The authors take full responsibility for the accuracy, interpretation, and final content of the manuscript and supplementary materials.

## 3. Results

The results describe the distribution of microbiological non-compliance in ready-to-eat (RTE) foods according to temporal, territorial, operational, and food-related characteristics.

### 3.1 Sample characterization

A total of 1,868 RTE food samples were analyzed, of which 1,846 were included in the inferential analyses. The temporal distribution showed an expansion of the surveillance system in more recent years, with lower representativeness in 2020. Samples were primarily obtained from food service establishments, followed by food operators and institutional settings, reflecting the distribution of samples collected through the official surveillance program. Geographically, the highest proportion of samples originated from Villavicencio, followed by intermediate municipalities. Regarding food matrices, ready-to-eat foods intended for immediate consumption predominated, particularly raw or highly handled foods such as juices and salads. Microbiological non-compliance was identified in 39.5% of the evaluated samples (Table 1).

**Table 1:** Characteristics of ready-to-eat food samples included in the official food safety surveillance system (n = 1,868). Data are presented as absolute (n) and relative (%) frequencies. Percentages were calculated using the total number of samples included in the surveillance system as the denominator. The microbiological assessment status includes all samples analyzed during the study period; samples without a valid microbiological assessment were excluded from subsequent inferential analyses. Municipalities, establishment types, and food matrices were grouped according to the categories used in the statistical analyses. Food matrices were classified according to their processing and handling characteristics. Superscripts indicate the corresponding analytical categories: a, departmental capital and largest urban center; b, intermediate municipalities; c, rural/dispersed municipalities; d, raw or highly handled foods; e, thermally processed foods; f, liquid preparations; g, dairy and sweet products; h, other less frequently sampled foods; i, food service; j, food operators; k, institutional establishments; l, distribution/logistics establishments; m, other establishments.

Category	Subcategory	n	%	Category	Subcategory	n	%
Temporal patterns	2017	203	10.9	Types of establishments	**Restaurants** ^i^	708	37.9
	2018	275	14.7		**Food operator** ^j^	282	15.1
	2019	204	10.9		**Cafeteria** ^i^	230	12.3
	2020	113	6.0		**Educational institution** ^k^	140	7.5
	2021	319	17.1		**Childcare center** ^k^	134	7.2
	2022	381	20.4		**Penitentiary center** ^k^	110	5.9
	2023	373	20.0		**Nursing home (elderly care facility)** ^k^	109	5.8
Municipal distribution	Villavicencio ^a^	598	32.0		**Supermarkets** ^l^	57	3.1
	Acacias ^b^	314	16.8		**Street food vendors** ^i^	30	1.6
	Puerto Gaitán ^b^	87	4.7		Hospitals ^k^	19	1.0
	Mesetas ^c^	77	4.1		**Public market** ^i^	17	0.9
	Granada ^b^	68	3.6		**Transport – vehicle** ^l^	10	0.5
	Cumaral ^b^	48	2.6		**Distributor** ^l^	8	0.4
	San Carlos G ^c^	46	2.5		**Military battalion** ^m^	7	0.4
	Cabuyaro ^c^	42	2.2		**Household** ^m^	7	0.4
	Puerto López ^b^	42	2.2	Type of food matrix	Juice ^d^	384	20.6
	El Castillo ^c^	36	1.9		Salad ^d^	305	16.3
	Fuentedeoro ^c^	36	1.9		Meat ^e^	174	9.3
	Mapiripán ^c^	36	1.9		Rice ^e^	163	8.7
	San Martín ^b^	36	1.9		Empanada ^e^	155	8.3
	Puerto Lleras ^c^	35	1.9		Broth ^f^	90	4.8
	Barranca Upía ^c^	34	1.8		Dessert ^g^	90	4.8
	Cubarral ^c^	34	1.8		Chicken ^e^	69	3.7
	Restrepo ^b^	33	1.8		Arepa ^e^	67	3.6
	Lejanías ^c^	32	1.7		Sausage ^e^	53	2.8
	El Calvario ^c^	30	1.6		Beans ^e^	48	2.6
	Guamal ^c^	29	1.6		Egg ^e^	40	2.1
	Castilla Nueva ^c^	28	1.5		Panela (unrefined cane sugar) ^g^	37	2.0
	El Dorado ^c^	26	1.4		Oatmeal ^f^	32	1.7
	Puerto Rico ^c^	26	1.4		Milk ^g^	31	1.7
	San Juanito ^c^	24	1.3		Soup ^f^	24	1.3
	Vistahermosa ^c^	23	1.2		Hamburger ^e^	22	1.2
	Puerto Concordia ^c^	17	0.9		Coffee/hot chocolate ^f^	14	0.7
	San Juan Arama ^c^	11	0.6		Stew ^e^	13	0.7
	Medina ^c^	6	0.3		Fish ^e^	13	0.7
	Guayabetal ^c^	5	0.3		Potato ^e^	12	0.6
	Paratebueno ^c^	4	0.2		Salpicón ^d^	10	0.5
	Uribe ^c^	3	0.2		Masato (fermented beverage) ^f^	5	0.3
	La Macarena ^c^	2	0.1		Custard ^g^	4	0.2
					Pasta ^e^	3	0.2
Microbiological assessment status	Microbiologically compliant	1108	59.3			Cheese ^h^	3	0.2
	Microbiologically non-compliant	738	39.5			Salt ^h^	3	0.2
	No valid microbiological assessment	22	1.2			Water ^h^	2	0.1
						Ham ^h^	2	0.1
Total observations: 14944 (100%).							

### 3.2 Microbiological profile of non-compliant ready-to-eat food samples

Microbiological non-compliance was identified in 39.5% of the evaluated samples. Among the microbiological parameters, total coliforms showed the highest proportion of non-compliance (29.0%), followed by aerobic mesophilic bacteria (12.7%) and *Escherichia coli* (12.3%). In contrast, foodborne pathogens were detected infrequently, including *Bacillus cereus* (2.2%), coagulase-positive *Staphylococcus* (1.8%), *Salmonella* spp. (0.2%), and *Listeria monocytogenes* (0%). Overall, most microbiological non-compliance events were attributable to exceedances of hygienic indicator criteria rather than to the detection of specific pathogens (Table 2).

**Table 2:** Microbiological profile of ready-to-eat food samples with valid microbiological assessment (n = 1,846). Data are presented as the number and proportion (%) of samples that did not meet the applicable microbiological criterion for each parameter. Percentages were calculated using the total number of samples with a valid microbiological assessment (n = 1,846) as the denominator. Ninety-five percent confidence intervals (95% CI) are reported for each proportion.

Indicator microorganism/pathogen	Microbiological non-compliance	(%)	95% CI
Total coliforms	535	29	26.6 - 30.6
Aerobic mesophilic bacteria	234	12.7	11-14
*E. coli*	227	12.3	10.7 -13.6
Molds & yeasts	154	8.3	7 - 9.5
*Bacillus cereus*	41	2.2	1.5 - 2.8
Coagulase-positive *Staphylococcus*	33	1.8	1.2 - 2.4
*Salmonella* spp.	3	0.2	0 - 0.3
*Listeria monocytogenes*	0	0	0 - 0.2

#### 3.2.1 Temporal patterns in the proportion of samples positive for microbiological parameters in ready-to-eat foods (2017–2023).

Fig 1 summarizes the temporal distribution of the proportion of samples testing positive for each microbiological parameter between 2017 and 2023. Hygiene indicators, particularly total coliforms, *Escherichia coli*, and aerobic mesophilic microorganisms, consistently showed higher observed proportions of positive samples than foodborne pathogens throughout the surveillance period. Total coliforms showed the highest observed proportions across years, ranging from 19.4% in 2021 to 38.2% in 2019. Several parameters showed relatively higher values in 2020 compared with their parameter-specific temporal distributions, including *Bacillus cereus*, aerobic mesophilic microorganisms, molds and yeasts, and coagulase-positive *Staphylococcus.* In contrast, Salmonella spp. and Listeria monocytogenes were infrequently detected throughout the surveillance period. The standardized color scale facilitates visualization of relative year-to-year variation within each microbiological parameter, while the percentages displayed in the cells represent the observed proportions of positive samples.

### 3.3 Bivariate association between contextual variables and microbiological non-compliance in ready-to-eat foods

The bivariate analysis (S2 Table) showed no significant differences in microbiological non-compliance according to sampling year. Although a higher proportion of non-compliant samples was observed in 2020, this difference did not reach statistical significance. In contrast, significant associations were identified for several operational variables. Establishment type was significantly associated with microbiological non-compliance. Food operators had higher odds of microbiological non-compliance, whereas food service establishments had lower odds. Institutional settings also showed higher odds of microbiological non-compliance, while distribution and logistics establishments were not significantly associated with the outcome. Food matrix type showed a strong association with microbiological non-compliance. Raw or highly handled foods had significantly higher odds of microbiological non-compliance than thermally processed foods. This association remained statistically significant in the multivariable analysis (Table 3). At the territorial level, significant differences were also observed. Samples collected in rural or dispersed municipalities had higher odds of microbiological non-compliance, whereas samples collected in Villavicencio had lower odds of microbiological non-compliance. Intermediate municipalities showed no significant association with microbiological non-compliance (S2 Table).

**Table 3:** Multivariable logistic regression model for factors associated with microbiological non-compliance in ready-to-eat foods (n = 1,846). Regression coefficients (B), standard errors, Wald statistics, p-values, odds ratios [OR], and 95% confidence intervals (95% CI) are presented. The model was adjusted for temporal variables (year 2020), territorial variables (rural/dispersed municipalities and Villavicencio), type of establishment (food service, food operator, and institutional), and food matrix type (raw or highly handled foods, liquid preparations, and dairy/sweet products), using thermally processed foods as the reference category. Odds ratios >1 indicate higher odds of microbiological non-compliance, whereas odds ratios <1 indicate lower odds of microbiological non-compliance. Statistical significance was defined as p < 0.05.

	B	Standard error	Wald	P-value	OR	95% CI	
						Lower	Upper
2020 (1)	0.577	0.221	6.85	0.009	1.781	1.156	2.744
Villavicencio (1)	0.208	0.148	1.985	0.159	1.231	0.922	1.644
Rural/dispersed municipalities (1)	0.27	0.132	4.174	0.041	1.31	1.011	1.698
Food service (1)	-0.27	0.273	0.98	0.322	0.763	0.447	1.303
Food operator (1)	0.073	0.3	0.059	0.808	1.075	0.598	1.935
Institutional (1)	-0.191	0.285	0.449	0.503	0.826	0.473	1.443
Raw or highly handled foods (1)	2.129	0.124	293.939	<0.001	8.403	6.588	10.718
Liquid preparations (1)	-0.138	0.229	0.366	0.545	0.871	0.556	1.363
Dairy/sweet products (1)	-0.368	0.233	2.498	0.114	0.692	0.439	1.092
Constant	-1.25	0.285	19.283	0	0.287		

#### 3.3.1 Patterns in the proportion of samples positive for microbiological parameters according to establishment type.

S1 Fig shows differences in the proportion of samples positive for microbiological parameters according to establishment type. Hygiene indicators, particularly total coliforms and *Escherichia coli,* predominated across most establishment categories, whereas foodborne pathogens were infrequently detected. Higher proportions of samples positive for hygiene indicators were observed in food operators, transportation-related establishments, street vendors, and some institutional settings. Aerobic mesophilic microorganisms, molds, and yeasts showed variable patterns across establishment types. In contrast, Salmonella spp. and Listeria monocytogenes were detected only occasionally. Household settings showed a different microbiological profile compared with other establishment categories, whereas distribution and logistics establishments generally showed lower proportions of positive samples for most microbiological parameters.

#### 3.3.2 Spatial distribution of the proportion of samples positive for microbiological parameters.

S2 Fig shows spatial variation in the proportion of samples positive for microbiological parameters across municipalities. Hygiene indicators, particularly total coliforms and *Escherichia coli,* predominated in most municipalities, whereas foodborne pathogens were infrequently detected throughout the study area. Higher proportions of samples positive for hygiene indicators were observed in several rural and dispersed municipalities, whereas urban municipalities generally showed intermediate proportions. Aerobic mesophilic microorganisms, molds, and yeasts showed variable spatial patterns. Municipalities with smaller numbers of samples exhibited greater variability in the observed proportions than municipalities with larger sample sizes

### 3.4 Exploratory multivariate analyses and multivariable logistic regression

#### 3.4.1 Exploratory multivariate analysis of microbiological profiles across municipalities.

Fig 2 presents a principal component analysis (PCA) exploring the relationships between municipalities and the microbiological parameters evaluated in ready-to-eat foods, explaining 57.9% of the total variance (PC1: 37.2%; PC2: 20.7%). The distribution of municipalities and microbiological parameters in the PCA space showed distinct exploratory patterns. Hygiene indicators, particularly total coliforms, *Escherichia coli*, aerobic mesophilic microorganisms, and molds and yeasts, were positioned relatively close to each other in the PCA space, whereas foodborne pathogens showed different patterns. Municipalities were distributed along the principal component axes according to their microbiological profiles, indicating variability in the distribution of microbiological parameters across the study area.

**Fig 2:**
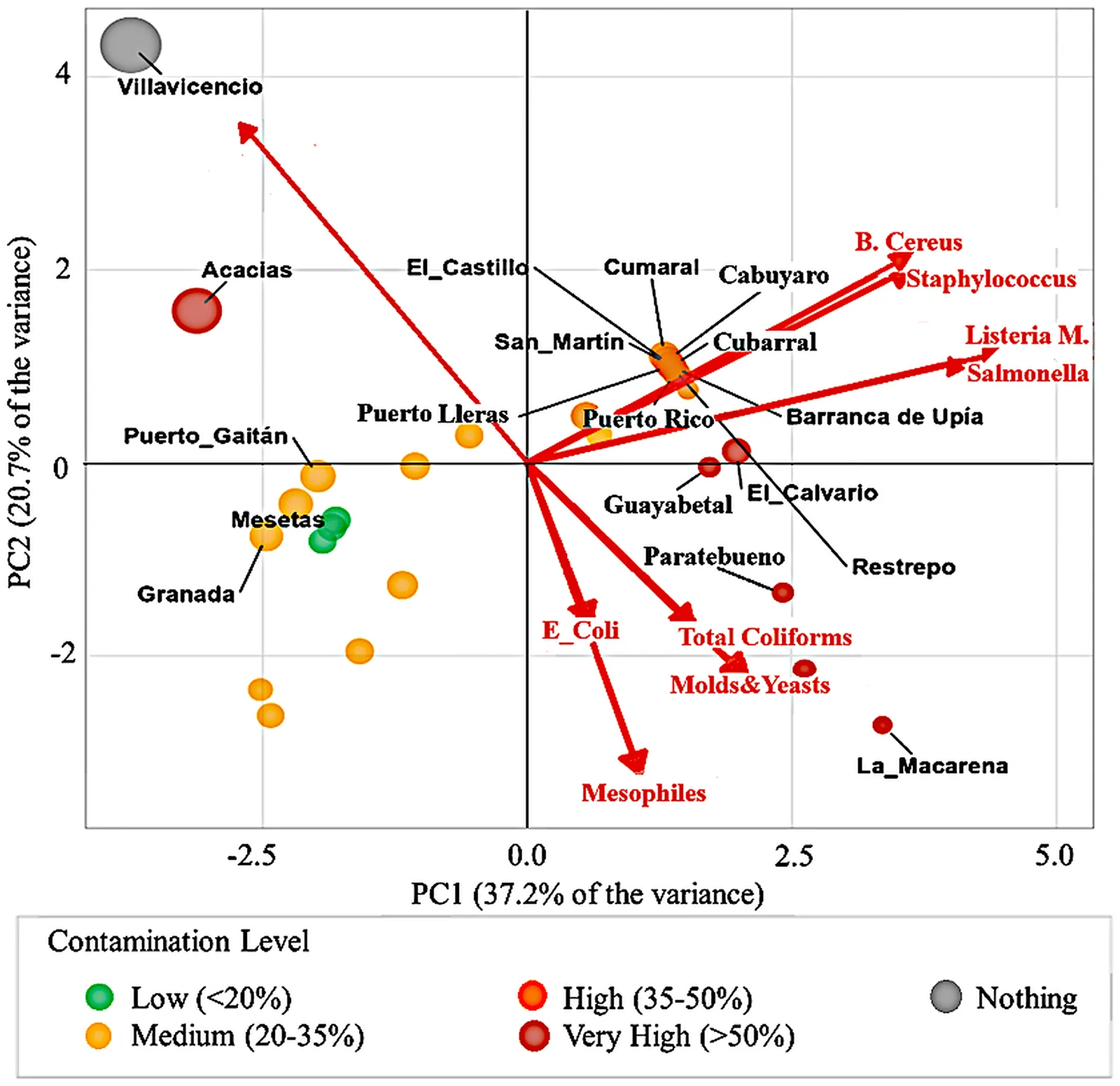
Exploratory principal component analysis of microbiological profiles across municipalities. Principal component analysis (PCA) showing the distribution of municipalities according to the microbiological parameters evaluated in ready-to-eat foods. PC1 and PC2 explained 37.2% and 20.7% of the total variance, respectively. Vectors represent the direction and contribution of the evaluated microbiological parameters, whereas municipalities are positioned according to similarities and differences in their microbiological profiles. The analysis was exploratory and was not interpreted as evidence of causal or microorganism-specific determinants of microbiological non-compliance.

#### 3.4.2 Exploratory multivariate analysis of microbiological profiles across food matrices.

Fig 3 presents a principal component analysis (PCA) exploring the relationships between food matrices and the evaluated microbiological parameters, explaining 64.7% of the total variance (PC1: 46.4%; PC2: 18.3%). The distribution of food matrices and microbiological parameters in the PCA space showed distinct exploratory patterns. Hygiene indicators, including total coliforms, *Escherichia coli*, aerobic mesophilic microorganisms, and molds and yeasts, were positioned relatively close to each other in the PCA space. These indicators were also positioned closer to some raw or highly handled food matrices, including juices, salads, and salpicon. In contrast, foodborne pathogens showed different patterns in the PCA space. Overall, food matrices exhibited heterogeneous microbiological profiles, indicating variability in the distribution of microbiological parameters according to food type.

**Fig 3:**
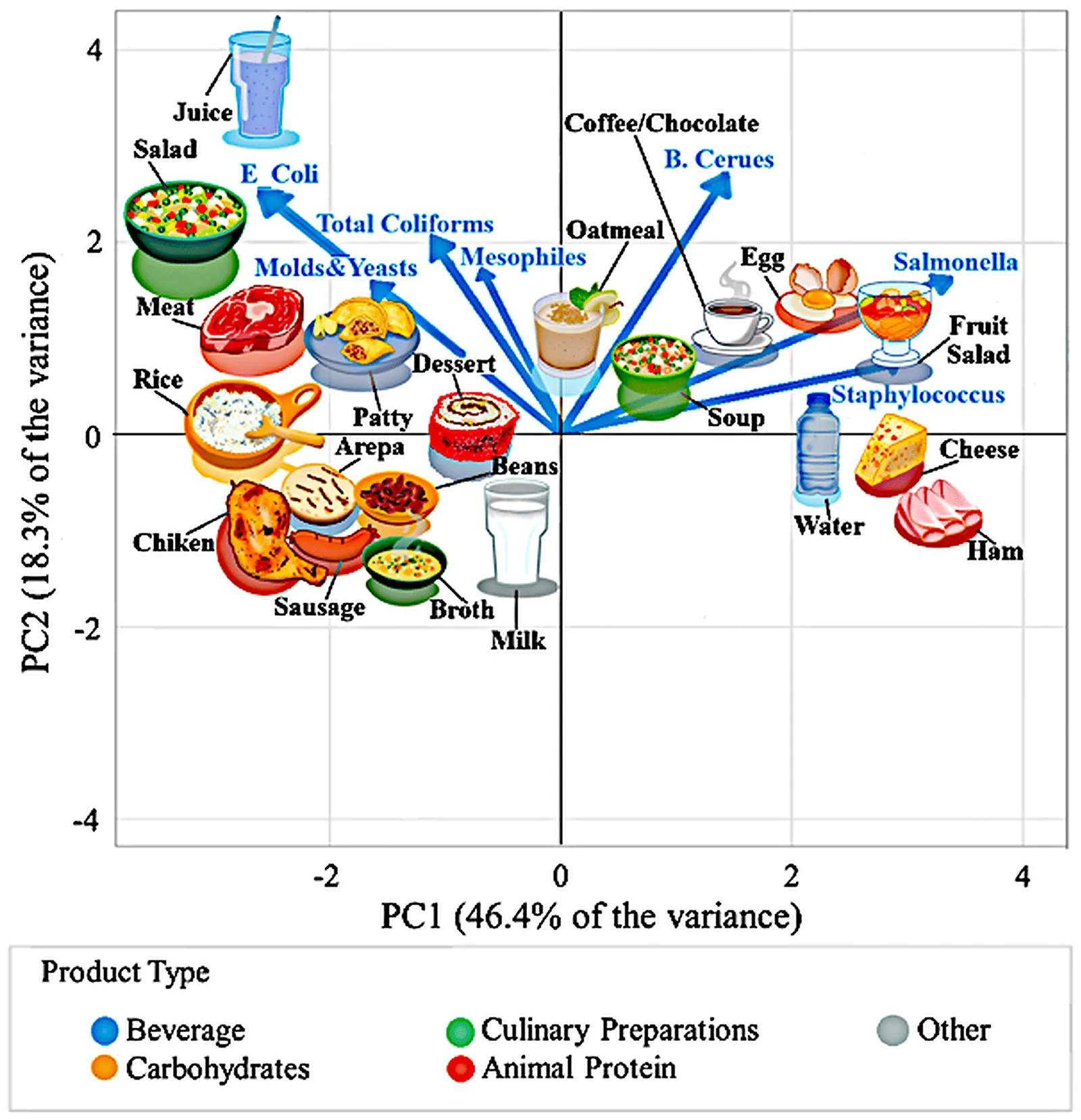
Exploratory principal component analysis of microbiological profiles across food matrices. Principal component analysis (PCA) showing the distribution of food matrices according to the microbiological parameters evaluated in ready-to-eat foods. PC1 and PC2 explained 46.4% and 18.3% of the total variance, respectively. Vectors represent the direction and contribution of the evaluated microbiological parameters, whereas food matrices are positioned according to similarities and differences in their microbiological profiles. The analysis was exploratory and was not interpreted as evidence of causal or microorganism-specific determinants of microbiological non-compliance.

#### 3.4.3 Multivariable logistic regression.

The multivariable logistic regression model identified several factors independently associated with microbiological non-compliance in ready-to-eat foods (Table 3). Samples collected in 2020 had higher odds of microbiological non-compliance than those collected during the reference period (OR = 1.78; 95% CI: 1.16–2.74; p = 0.009). Likewise, samples from rural or dispersed municipalities had higher odds of microbiological non-compliance than those from intermediate municipalities, which constituted the reference category (OR = 1.31; 95% CI: 1.01–1.70; p = 0.041). Villavicencio was not significantly associated with microbiological non-compliance compared with intermediate municipalities (OR = 1.23; 95% CI: 0.92–1.64; p = 0.159). Regarding food matrix type, raw or highly handled foods had substantially higher odds of microbiological non-compliance than thermally processed foods (OR = 8.40; 95% CI: 6.59–10.72; p < 0.001). In contrast, liquid preparations (OR = 0.87; 95% CI: 0.56–1.36; p = 0.545) and dairy and sweet products (OR = 0.69; 95% CI: 0.44–1.09; p = 0.114) were not significantly associated with microbiological non-compliance.

Establishment type was not significantly associated with microbiological non-compliance in the multivariable model. Food service establishments (OR = 0.76; 95% CI: 0.45–1.30; p = 0.322), food operators (OR = 1.08; 95% CI: 0.60–1.94; p = 0.808), and institutional settings (OR = 0.83; 95% CI: 0.47–1.44; p = 0.503) showed no statistically significant associations. The model showed adequate goodness-of-fit according to the Hosmer–Lemeshow test (χ² = 6.355; p = 0.608). The Cox and Snell R^2^ was 0.226 and the Nagelkerke R^2^ was 0.306. Overall classification accuracy was 75.8%, with 81.9% of compliant samples and 66.5% of non-compliant samples correctly classified.

## 4. Discusión

The findings of this study indicate that microbiological non-compliance in ready-to-eat (RTE) foods was primarily associated with food matrix type, territorial characteristics, and sampling year, whereas foodborne pathogens were infrequently detected.

### 4.1 Main findings and contributions of the study

This study represents an integrated analytical evaluation based on official surveillance data from the Colombian Orinoquía, integrating descriptive, multivariate, and inferential evidence within a single analytical framework. The magnitude of the observed microbiological non-compliance suggests persistent challenges in hygiene and handling of RTE foods, consistent with reports from tropical and middle-income settings, where food safety remains an important public health challenge [13–16]. Beyond quantifying microbiological non-compliance, the main contribution of this study lies in identifying temporal, territorial, and food-related factors associated with failure to comply with microbiological standards using seven years of official surveillance data.

### 4.2 Predominance of hygiene indicators and public health significance

The predominance of hygiene indicators, such as total coliforms, *Escherichia coli*, and mesophilic microorganisms, suggests that most microbiological non-compliance events were attributable to failures in hygienic indicator criteria rather than to the detection of foodborne pathogens. The low frequency of foodborne pathogens should not be interpreted as evidence of the absence of potential public health concern. Rather, it indicates that, within this surveillance dataset, most microbiological non-compliance events were related to hygienic indicators rather than pathogen detection. These findings are consistent with studies describing hygienic indicators as useful markers of food handling and process hygiene because they reflect multiple potential sources of contamination, including water quality, handling practices, environmental surfaces, and temperature control [12,17,18].

### 4.3. Implications for food safety surveillance in tropical LMIC settings

The high proportion of microbiological non-compliance observed in this study (39.5%) mirrors findings reported in other tropical regions, such as parts of Southeast Asia and Sub-Saharan Africa [19–21], where environmental temperature and humidity have been associated with increased microbial proliferation in RTE foods [22]. In our study, samples collected from rural and dispersed municipalities showed significantly higher odds of microbiological non-compliance (OR = 1.31), indicating geographic differences in microbiological non-compliance. Although this study did not directly evaluate the underlying causes of these differences, previous studies have suggested that factors such as access to safe water, cold-chain infrastructure, and food handling conditions may contribute to geographical disparities in food safety performance in LMICs [1,23]. These findings support the importance of strengthening risk-based food safety surveillance and targeted interventions in tropical settings to improve compliance with microbiological standards and contribute to broader public health and food safety goals.

### 4.4 Integration of descriptive, multivariate, and inferential patterns

Descriptive analyses (heatmaps) revealed consistent heterogeneity in the proportion of samples positive for microbiological parameters across temporal, geographical, and operational dimensions, with a systematic predominance of hygiene indicators across most contexts. This pattern was consistent with the exploratory PCA, in which hygiene indicators were positioned relatively close to each other and showed patterns of association with raw or highly handled food matrices, such as salads and juices.

The collinearity observed among total coliforms, *Escherichia coli*, mesophilic microorganisms, and molds/yeasts suggests that these indicators reflect common patterns of hygienic quality rather than independent contamination events. This finding is consistent with conceptual models that interpret microbiological indicators as integrative markers of hygienic conditions within food production and handling processes [2]. In contrast, foodborne pathogens showed a dispersed distribution and were detected at low frequencies across the surveillance dataset.

From a territorial perspective, the differentiation between municipalities observed in PCA and heatmaps suggests geographic variability in microbiological occurrence, which may be related to differences previously described in sanitation infrastructure, access to safe water, and food handling conditions [13,15]. Consistently, the descriptive analysis by establishment type showed higher proportions of samples positive for hygiene indicators in transportation-related establishments and informal food vending settings.

Within this framework, the multivariable logistic regression model provides inferential evidence supporting these patterns, demonstrating independent associations between microbiological non-compliance and temporal, territorial, and food matrix-related variables. The higher odds observed in 2020 may reflect contextual or operational changes occurring during that period; however, the specific causes could not be evaluated in the present study. This type of behavior has been described in epidemiological surveillance systems, where modifications in sampling intensity, selection criteria, or external conditions can influence event detection and observed estimates [24]. Similarly, the higher odds of microbiological non-compliance observed in rural or dispersed municipalities are consistent with previous studies reporting geographical differences in food safety performance in LMICs, although the present study did not directly evaluate the underlying causes of these differences [13,15].

A central finding of this study is the strong and consistent association observed for raw or highly handled food matrices in the adjusted model. Unlike the initial model specification, where collinearity generated unstable and potentially misleading estimates, the reparametrized model revealed that raw or highly handled foods—characterized by intensive handling and absence of terminal heat treatment—showed the strongest independent association with microbiological non-compliance. The magnitude of the observed association (OR > 8) indicates that food matrix type was the variable most strongly associated with microbiological non-compliance in the final model. This result is fully consistent with the descriptive and multivariate analyses, and with evidence indicating that raw or highly handled foods are more susceptible to contamination due to the absence of a final microbial inactivation step and the presence of multiple critical control points along the production chain [4,12]. In contrast, the lack of association observed for liquid preparations and dairy/sweet products after adjustment suggests that differences observed in unadjusted analyses may have been explained by confounding factors controlled for in the adjusted model. This phenomenon has been widely described in epidemiology, where apparent associations may change substantially after controlling for contextual variables [25]

Notably, variables related to type of establishment and urban location did not retain statistical significance in the adjusted model, indicating that microbiological non-compliance was not independently associated with these variables after adjustment. This finding is consistent with contemporary approaches advocating a shift from establishment-centered models toward food system-based analytical frameworks [2].

Overall, the integration of descriptive, exploratory, and inferential analyses showed consistent patterns of microbiological occurrence and identified temporal, territorial, and food matrix-related factors independently associated with microbiological non-compliance. Hygienic indicators predominated throughout the surveillance period, whereas foodborne pathogens were detected only infrequently.

Consequently, this study extends descriptive surveillance by identifying factors independently associated with microbiological non-compliance using seven years of official food safety surveillance data. These findings provide evidence to support risk-based food safety surveillance and prioritization strategies in tropical low- and middle-income settings.

### 4.5 Public health implications and risk-based surveillance

The findings support the need to transition toward risk-based food safety surveillance approaches, prioritizing interventions targeting food matrices and geographical areas with higher proportions of microbiological non-compliance. Likewise, the observed territorial heterogeneity supports the consideration of differentiated surveillance strategies tailored to specific contexts, to optimize resource allocation and maximize the impact of public health interventions. In this context, microbiological indicators play a central role as early monitoring tools for detecting changes in hygienic quality and process control in complex food systems [12].

### 4.6 Strengths and limitations

Among the main strengths of this study is the use of data derived from an official surveillance system with broad and sustained coverage, which allowed the integration of multiple analytical approaches within a single framework. However, the study has limitations inherent to the use of secondary data, including potential variations in sampling criteria and data recording quality. Additionally, non-probabilistic sampling may introduce representativeness bias, particularly in rural settings. The absence of variables related to food handling practices, water quality, sanitation infrastructure, and environmental conditions precluded direct evaluation of the mechanisms underlying the observed associations. Finally, quantitative microbial concentrations were not consistently available in a standardized format across all surveillance years and microbiological parameters; therefore, the study could not evaluate microbial loads expressed as log CFU/g or log CFU/mL

### 4.7 Future perspectives

Future research should further investigate microorganism-specific patterns in microbiological findings and factors associated with microbiological non-compliance, as well as the development of quantitative microbial risk assessment models. These approaches would contribute to a better understanding of microbiological contamination patterns and support the development of more targeted food safety surveillance and control strategies.

## 5. Conclusion

This study identified factors associated with microbiological non-compliance in ready-to-eat (RTE) foods. By integrating multi-year surveillance data with multivariate approaches, the findings showed that microbiological non-compliance was primarily associated with food matrix type, territorial characteristics, and sampling year, whereas foodborne pathogens were detected infrequently. The strong association identified for raw or highly handled food matrices, together with the association observed for rural or dispersed municipalities, supports the prioritization of risk-based food safety surveillance strategies focused on food categories and geographical areas with higher proportions of microbiological non-compliance. These findings provide evidence to support surveillance and control strategies in tropical low- and middle-income countries. From a public health perspective, the predominance of hygiene indicators suggests that microbiological surveillance should continue to incorporate indicators of hygienic quality and process control in addition to pathogen detection. In this context, microbiological indicators provide useful information for monitoring compliance with microbiological standards and supporting evidence-based decision-making.

Overall, this study demonstrates the value of integrating official surveillance data with multivariable analytical approaches to identify factors associated with microbiological non-compliance and to support risk-based food safety surveillance strategies, particularly in tropical low- and middle-income settings.

## Supporting information

S1 Table Microbiological analytical methods used for routine food safety surveillance in the Public Health Laboratory. The table summarizes the analytical methods, reference standards, culture media or analytical systems, incubation conditions, laboratory procedures, reporting units, and detection limits used for each microbiological parameter. Abbreviations: AOAC, Association of Official Analytical Collaboration; CFU, colony-forming units; FDA, U.S. Food and Drug Administration; ICMSF, International Commission on Microbiological Specifications for Foods; ISO, International Organization for Standardization; MPN, most probable number; PCA, Plate Count Agar; TEMPO, automated microbiological enumeration system; XLD, Xylose Lysine Deoxycholate agar.
(DOCX)

S2 Table Bivariate associations between contextual variables and microbiological non-compliance in ready-to-eat foods (n = 1,846). Associations were assessed using Pearson’s chi-square test. Odds ratios (OR) and 95% confidence intervals (95% CI) are reported. Percentages were calculated by row. “Yes” indicates the presence of the evaluated condition, whereas “No” represents the reference category. Statistical significance was defined as p < 0.05.
(DOCX)

S1 Fig Distribution of the proportion of samples positive for microbiological parameters according to establishment type. Cell values represent the observed proportion of positive samples (%) for each microbiological parameter within each establishment category. Color intensity reflects the magnitude of the observed proportion of positive samples. This heatmap was used for descriptive and exploratory purposes and was not standardized using z-scores.
(TIF)

S2 Fig Spatial distribution of the proportion of samples positive for microbiological parameters across municipalities. Cell values represent the observed proportion of positive samples (%) for each microbiological parameter within each municipality; sample sizes are shown in parentheses where applicable. Color intensity reflects the magnitude of the observed proportion of positive samples. This heatmap was used for descriptive and exploratory purposes and was not standardized using z-scores.
(TIF)

S1 File Graphical abstract. Overview of the study design, analytical framework, microbiological non-compliance profile, and factors associated with microbiological non-compliance in ready-to-eat foods in the Meta Department, Colombia, 2017–2023. The South America base map was adapted from Wikimedia Commons (CC0 1.0 Universal Public Domain Dedication; https://commons.wikimedia.org/wiki/File:South_America_blank_map.svg). The boundaries of the Meta Department were derived from the official geo-statistical cartography of the National Administrative Department of Statistics (DANE), Colombia (https://geoportal.dane.gov.co/servicios/descarga-y-metadatos/datos-geoestadisticos/?cod=111). Data are distributed under the DANE Geoportal terms of use compatible with the Creative Commons Attribution 4.0 International License (CC BY 4.0). Some icons were obtained from Openclipart (CC0 1.0 Public Domain). All remaining graphical elements and representations of study results were created by the authors.
(TIF)

## References

[1] WHO. Estimating the burden of foodborne diseases. https://www.who.int/activities/estimating-the-burden-of-foodborne-diseases. 2020.

[2] FAO, WHO. World Food Safety Day 2021. FAO. 2021. doi: 10.4060/cb6125en

[3] Lima Tribst AA, de Souza Sant’Ana S, de Massaguer PR. Review: Microbiological quality and safety of fruit juices—past, present and future perspectives. Crit Rev Microbiol. 2009;35(4):310–39. doi: 10.3109/10408410903241428 19863382

[4] Makinde OM, Ayeni KI, Sulyok M, Krska R, Adeleke RA, Ezekiel CN. Microbiological safety of ready-to-eat foods in low- and middle-income countries: A comprehensive 10-year (2009 to 2018) review. Compr Rev Food Sci Food Saf. 2020;19(2):703–32. doi: 10.1111/1541-4337.12533 33325184

[5] Murray K, Wu F, Shi J, Jun Xue S, Warriner K. Challenges in the microbiological food safety of fresh produce: Limitations of post-harvest washing and the need for alternative interventions. Food Quality and Safety. 2017;1(4):289–301. doi: 10.1093/fqsafe/fyx027

[6] Tchonkouang RD, Onyeaka H, Nkoutchou H. Assessing the vulnerability of food supply chains to climate change-induced disruptions. Sci Total Environ. 2024;920:171047. doi: 10.1016/j.scitotenv.2024.171047 38373458

[7] Elbehiry A, Marzouk E, Abalkhail A, Edrees HM, Ellethy AT, Almuzaini AM, et al. Microbial food safety and antimicrobial resistance in foods: A dual threat to public health. Microorganisms. 2025;13(7):1592. doi: 10.3390/microorganisms13071592 40732101 PMC12299635

[8] Mazaheri T, Cervantes-Huamán BRH, Bermúdez-Capdevila M, Ripolles-Avila C, Rodríguez-Jerez JJ. Listeria monocytogenes biofilms in the food industry: Is the current hygiene program sufficient to combat the persistence of the pathogen?. Microorganisms. 2021;9(1):181. doi: 10.3390/microorganisms9010181 33467747 PMC7830665

[9] Nguyen AT, Tallent SM. Screening food for *Bacillus cereus* toxins using whole genome sequencing. Food Microbiol. 2019;78:164–70. doi: 10.1016/j.fm.2018.10.008 30497598

[10] Hölzel CS, Tetens JL, Schwaiger K. Unraveling the role of vegetables in spreading antimicrobial-resistant bacteria: A need for quantitative risk assessment. Foodborne Pathog Dis. 2018;15(11):671–88. doi: 10.1089/fpd.2018.2501 30444697 PMC6247988

[11] Michel M, Eldridge AL, Hartmann C, Klassen P, Ingram J, Meijer GW. Benefits and challenges of food processing in the context of food systems, value chains and sustainable development goals. Trends Food Sci Technol. 2024;153:104703. doi: 10.1016/j.tifs.2024.104703

[12] Koutsoumanis K, Ordóñez AA, Bolton D, Bover‐Cid S, Chemaly M, De Cesare A. Microbiological hazards associated with the use of water in the post‐harvest handling and processing operations of fresh and frozen fruits, vegetables and herbs (ffFVHs). Part 1 (outbreak data analysis, literature review and stakeholder questionnaire). EFSA Journal. 2023;21. doi: 10.2903/j.efsa.2023.8332 PMC10623241 37928944

[13] Grace D. Food Safety in low and middle income countries. Int J Environ Res Public Health. 2015;12(9):10490–507. doi: 10.3390/ijerph120910490 26343693 PMC4586623

[14] Havelaar AH, Kirk MD, Torgerson PR, Gibb HJ, Hald T, Lake RJ, et al. World Health Organization global estimates and regional comparisons of the burden of foodborne disease in 2010. PLoS Med. 2015;12:e1001923. doi: 10.1371/journal.pmed.1001923 PMC4668832 26633896

[15] Kirk MD, Pires SM, Black RE, Caipo M, Crump JA, Devleesschauwer B. World Health Organization estimates of the global and regional disease burden of 22 foodborne bacterial, protozoal, and viral diseases, 2010: A data synthesis. PLoS Med. 2015;12:e1001921. doi: 10.1371/journal.pmed.1001921 PMC4668831 26633831

[16] Hald T, Aspinall W, Devleesschauwer B, Cooke R, Corrigan T, Havelaar AH, et al. World Health Organization estimates of the relative contributions of food to the burden of disease due to selected foodborne hazards: A structured expert elicitation. PLoS One. 2016;11(1):e0145839. doi: 10.1371/journal.pone.0145839 26784029 PMC4718673

[17] Mengistu DA, Belami DD, Tefera AA, Alemeshet Asefa Y. Bacteriological quality and public health risk of ready-to-eat foods in developing countries: Systematic review and meta analysis. Microbiol Insights. 2022;15:11786361221113916. doi: 10.1177/11786361221113916 35898690 PMC9310325

[18] Pariza T, Cho MJ. Food safety in Latin American informal food establishments. Front Sustain. 2024;4. doi: 10.3389/frsus.2023.1325060

[19] Zheng Z, Ye L, Xiong W, Hu Q, Chen K, Sun R, et al. Prevalence and genomic characterization of the *Bacillus cereus* group strains contamination in food products in Southern China. Sci Total Environ. 2024;921:170903. doi: 10.1016/j.scitotenv.2024.170903 38354793

[20] Othman NM. Food safety in Southeast Asia: Challenges facing the region. Asian J Agric Dev. 2007;4(2):83–92. doi: 10.37801/ajad2007.4.2.6

[21] Christiana Cudjoe D, Balali GI, Titus OO, Osafo R, Taufiq M. Food safety in sub-Sahara Africa, An insight into Ghana and Nigeria. Environ Health Insights. 2022;16:11786302221142484. doi: 10.1177/11786302221142484 36530486 PMC9755555

[22] Duchenne-Moutien RA, Neetoo H. Climate change and emerging food safety issues: A review. J Food Prot. 2021;84:1884–97. doi: 10.4315/JFP-21-141 34185849

[23] Jaffee S, Henson S, Unnevehr L, Grace D, Cassou E. The safe food imperative: Accelerating progress in low- and middle-income countries. Washington, DC: World Bank. 2019. doi: 10.1596/978-1-4648-1345-0

[24] Fewell Z, Davey Smith G, Sterne JAC. The impact of residual and unmeasured confounding in epidemiologic studies: A simulation study. Am J Epidemiol. 2007;166(6):646–55. doi: 10.1093/aje/kwm165 17615092

[25] Delgado-Rodríguez M, Llorca J. Bias: Table 1. J Epidemiol Community Health. 2004;58:635–41. doi: 10.1136/jech.2003.008466 PMC1732856 15252064

