# Supplementary material for: Factors associated with microbiological non-compliance in ready-to-eat foods: A seven-year analysis of official food safety surveillance data in a tropical low- and middle-income country
Source: PLOS Glob Public Health. 2026 Oct 5;6(10):e0006492. doi: 10.1371/journal.pgph.0006492 (PMC13637920; doi:10.1371/journal.pgph.0006492)
Supplement: Supplementary file 1 [file pgph.0006492.s001.docx]

| **Supplementary Table S1. Microbiological analytical methods used for routine food safety surveillance in the Public Health Laboratory** | | | | | | |
| --- | --- | --- | --- | --- | --- | --- |
| **Microbiological parameter** | **Analytical**  **method** | **Reference standard** | **Culture medium / System** | **Incubation conditions** | **Laboratory procedure** | **Reporting unit / Detection limit** |
| Aerobic mesophilic | Plate count  (color indicator SimPlate) | ISO 4833:2003 | Plate Count Agar (PCA) | 35°C | P-SA-80 | <1 UFC |
|  | Automated TEMPO® AC |  | TEMPO® AC | 30 °C | P-SA-201 | <10 CFU/g or CFU/mL |
| Total coliforms | Color indicator plate count (EC-  SimPlate) | AOAC 2005.03;  AOAC 966.24 | EC-SimPlate | 35°C | P-SA-79 | < 1 UFC |
|  | Automated TEMPO® CC |  | TEMPO® CC |  | P-SA-202 | < 10 CFU/g or CFU/mL |
| *Escherichia coli* | Color indicator plate count (EC-SimPlate). | AOAC 2005.03; AOAC 966.23; AOAC 966.24 | EC-SimPlate | 35°C | P-SA-79 | < 1 UFC |
|  | Automated TEMPO® EC |  | TEMPO® EC | 37 °C | P-SA-200 | < 10 CFU/g or CFU/mL |
| Molds and yeasts | Plate count | ISO 21527  ICMSF | OGY agar | 25°C | P-SA-203 | < 10 CFU/g or CFU/mL |
|  | Automated TEMPO® YM |  | TEMPO® YM | 25°C |  |  |
| *Bacillus cereus* | Horizontal plate count method MYP | ISO 7932 | MYP agar | 30°C | P-SA-77 | < 100 CFU/g or CFU/mL |
|  | Automated TEMPO® BC |  | TEMPO® BC |  | P-SA-195 |  |
| Coagulase-positive *Staphylococcus* | Horizontal plate count method | ISO 6888-2 | Baird-Parker agar | 37°C | P-SA-78; | < 100 CFU/g or CFU/mL |
|  | Automated TEMPO® STA |  | TEMPO® STA |  | P-SA-194 |  |
| *Salmonella* spp. | Horizontal detection method. | ISO 6579:2017 | XLD agar and Bismuth sulfite (BS) | XLD: 37°C  BS: 35°C | P-SA-64 | Presence/absence in 25 g or 25 mL |
|  | PCR BAX |  |  | 37°C | P-SA-66 |  |
| *Listeria monocytogenes* | Horizontal detection method | FDA reference method | PALCAM agar and Oxford agar | 37°C | P-SA-65 | Presence/absence in 25 g or 25 mL |
|  | PCR BAX |  |  | 55 °C | P-SA-67 |  |

**Table S1. Microbiological analytical methods used for routine food safety surveillance in the Public Health Laboratory.** The table summarizes the analytical methods, reference standards, culture media or analytical systems, incubation conditions, laboratory procedures, reporting units, and detection limits used for each microbiological parameter. Abbreviations: AOAC, Association of Official Analytical Collaboration; CFU, colony-forming units; FDA, U.S. Food and Drug Administration; ICMSF, International Commission on Microbiological Specifications for Foods; ISO, International Organization for Standardization; MPN, most probable number; PCA, Plate Count Agar; TEMPO®, automated microbiological enumeration system; XLD, Xylose Lysine Deoxycholate agar.
