# Supplementary material for: Factors associated with microbiological non-compliance in ready-to-eat foods: A seven-year analysis of official food safety surveillance data in a tropical low- and middle-income country
Source: PLOS Glob Public Health. 2026 Oct 5;6(10):e0006492. doi: 10.1371/journal.pgph.0006492 (PMC13637920; doi:10.1371/journal.pgph.0006492)
Supplement: Supplementary file 2 [file pgph.0006492.s002.docx]

| **Table S2. Bivariate associations between contextual variables and microbiological non-compliance in ready-to-eat foods** | | | | | | | | |
| --- | --- | --- | --- | --- | --- | --- | --- | --- |
| Associated variables | | Microbiological non-compliance | | Pearson Chi-Square | p-value | OR | 95% CI | |
|  |  | No n (%) | Yes n (%) |  |  |  | Lower | Upper |
| Temporal patterns | | | | | | | | |
| 2017-2019 | No | 710(60) | 473(40) | 0.00 | 0.996 | 0.99 | 0.82 | 1.21 |
|  | Yes | 398(60) | 265(40) |  |  |  |  |  |
| 2020 | No | 1049(60.5) | 684(39.5) | 3.05 | 0.080 | 1.40 | 0.95 | 2.05 |
|  | Yes | 59(52.2) | 54(47.8) |  |  |  |  |  |
| 2021-2023 | No | 457(58.9) | 319(41.1) | 0.71 | 0.399 | 0.92 | 0.76 | 1.11 |
|  | Yes | 651(60.8) | 419(39.2) |  |  |  |  |  |
| Types of establishments | | | | | | | | |
| Food service | No | 469(53.7) | 404(46.3) | 27.38 | < 0.001 | 0.60 | 0.50 | 0.73 |
|  | Yes | 639(65.7) | 334(34.3) |  |  |  |  |  |
| Food operator | No | 981(62.7) | 584(37.3) | 30.36 | < 0.001 | 2.03 | 1.57 | 2.63 |
|  | Yes | 127(45.2) | 154(54.8) |  |  |  |  |  |
| Institutional | No | 825(61.4) | 518(38.6) | 4.07 | 0.044 | 1.23 | 1.00 | 1.52 |
|  | Yes | 283(56.3) | 220(43.7) |  |  |  |  |  |
| Distribution/logistic | No | 1058(59.7) | 713(40.3) | 1.43 | 0.230 | 0.74 | 0.45 | 1.21 |
|  | Yes | 59(66.7) | 25(33.3) |  |  |  |  |  |
| Type of food matrix | | | | | | | | |
| Thermally processed foods | No | 453(45.2) | 549(54.8) | 200.3 | < 0.001 | 0.23 | 0.19 | 0.29 |
|  | Yes | 655(77.6) | 189(22.4) |  |  |  |  |  |
| Raw or highly handled foods | No | 908(78.6) | 247(21.4) | 444.5 | < 0.001 | 9.02 | 7.26 | 11.2 |
|  | Yes | 200(28.9) | 491(71.1) |  |  |  |  |  |
| Liquid preparations | No | 997(58.4) | 710(41.6) | 24.6 | < 0.001 | 0.35 | 0.23 | 0.54 |
|  | Yes | 111(79.9) | 28(20.1) |  |  |  |  |  |
| Dairy/sweet products | No | 971(57.8) | 710(42.2) | 39.97 | < 0.001 | 0.28 | 0.18 | 0.42 |
|  | Yes | 137(83) | 28(17) |  |  |  |  |  |
| Municipal distribution | | | | | | | | |
| Villavicencio | No | 708(55.8) | 560(44.2) | 29.56 | < 0.001 | 0.56 | 0.45 | 0.69 |
|  | Yes | 400(69.2) | 178(30.8) |  |  |  |  |  |
| Intermediate urban municipalities | No | 733(60.1) | 487(39.9) | 0.005 | 0.941 | 1.00 | 0.82 | 1.22 |
|  | Yes | 375(59.9) | 251(40.1) |  |  |  |  |  |
| Rural/dispersed municipalities | No | 775(64.4) | 429(35.6) | 27.26 | < 0.001 | 1.67 | 1.38 | 2.03 |
|  | Yes | 333(51.9) | 309(48.1) |  |  |  |  |  |

**Table S2. Bivariate associations between contextual variables and microbiological non-compliance in ready-to-eat foods (n = 1,846).** Associations were assessed using Pearson’s chi-square test. Odds ratios (OR) and 95% confidence intervals (95% CI) are reported. Percentages were calculated by row. “Yes” indicates the presence of the evaluated condition, whereas “No” represents the reference category. Statistical significance was defined as p < 0.05
